# Supplementary material for: Broadly neutralizing plasma antibodies effective against autologous circulating viruses in infants with multivariant HIV-1 infection
Source: Nat Commun. 2020 Sep 2;11:4409. doi: 10.1038/s41467-020-18225-x (PMC7468291; doi:10.1038/s41467-020-18225-x)
Supplement: Supplementary file 1 — Supplementary Information [file 41467_2020_18225_MOESM1_ESM.pdf]

Supplementary Information for

Broadly neutralizing plasma antibodies effective against autologous circulating viruses in infants with multivariant HIV-1 infection.

Mishra et al.

a

Infant ID	Age (M)	C								% Breadth	Geometric Mean Titres
		25710	25711	1428	16055	330.16.E6	SV21A	ST5B	329.14.B1		
AIIMS704	12	324	321	216	162	221	314	297	201	100	250
AIIMS706	9	209	189	254	138	152	369	164	164	100	195
AIIMS709	14	244	281	331	134	224	101	201	171	100	192
AIIMS743	14	357	227	291	113	247	101	252	204	100	207
AIIMS731	9	268	86	124	61	89	97	249	249	100	135
AIIMS719	12	321	310	258	136	112	164	102	118	100	178
AIIMS736	9	421	160	235	103	214	<50	<50	150	75	193
AIIMS744	14	297	89	103	117	144	118	<50	161	88	136
AIIMS732	7	112	110	259	141	137	132	94	112	100	139
AIIMS738	11	128	<50	<50	84	163	102	149	114	75	120
AIIMS721	24	181	66	114	88	162	164	<50	87	87	116
AIIMS703	9	114	247	88	74	84	78	<50	86	87	100
AIIMS705	6	158	87	118	72	87	81	<50	<50	75	97
AIIMS707	24	126	176	<50	<50	<50	91	146	<50	50	131
AIIMS733	12	254	<50	177	<50	264	<50	<50	<50	38	228
AIIMS742	19	114	<50	247	89	110	<50	<50	58	63	110
AIIMS716	24	102	<50	<50	<50	156	64	<50	<50	38	101
AIIMS720	9	135	<50	<50	<50	77	91	<50	<50	38	98
AIIMS702	24	89	<50	147	<50	88	82	<50	<50	50	99
AIIMS727	8	112	157	88	<50	105	<50	<50	<50	50	113
AIIMS735	8	118	138	68	<50	98	<50	<50	<50	50	102
AIIMS741	9	186	<50	214	<50	88	<50	<50	<50	38	152
AIIMS711	7	174	<50	152	64	<50	74	<50	<50	50	106
AIIMS717	8	115	97	<50	86	<50	<50	<50	<50	38	99
AIIMS718	11	116	<50	73	89	<50	<50	<50	<50	38	91
AIIMS712	19	89	<50	141	<50	<50	<50	<50	<50	25	112
AIIMS737	8	76	<50	186	<50	<50	<50	<50	<50	25	119
AIIMS747	7	78	<50	<50	<50	<50	<50	<50	<50	13	78
AIIMS713	12	91	<50	86	<50	<50	<50	<50	<50	25	88
AIIMS715	8	98	74	<50	<50	<50	<50	<50	<50	25	85
AIIMS701	24	91	<50	<50	<50	<50	<50	<50	<50	13	91
AIIMS722	13	98	<50	<50	<50	<50	<50	<50	<50	13	98
AIIMS723	12	89	<50	<50	<50	<50	<50	<50	<50	13	89
AIIMS724	14	78	<50	<50	<50	<50	<50	<50	<50	13	78
AIIMS726	18	67	<50	<50	<50	<50	<50	<50	<50	13	67
AIIMS708	18	<50	<50	<50	<50	<50	<50	<50	<50	0	0
AIIMS714	20	<50	<50	<50	<50	<50	<50	<50	<50	0	0
AIIMS710	6	<50	<50	<50	<50	<50	<50	<50	<50	0	0
AIIMS725	9	<50	<50	<50	<50	<50	<50	<50	<50	0	0
AIIMS728	8	<50	<50	<50	<50	<50	<50	<50	<50	0	0
AIIMS729	14	<50	<50	<50	<50	<50	<50	<50	<50	0	0
AIIMS730	18	<50	<50	<50	<50	<50	<50	<50	<50	0	0
AIIMS748	9	<50	<50	<50	<50	<50	<50	<50	<50	0	0
AIIMS734	24	<50	<50	<50	<50	<50	<50	<50	<50	0	0
AIIMS739	17	<50	<50	<50	<50	<50	<50	<50	<50	0	0
AIIMS740	24	<50	<50	<50	<50	<50	<50	<50	<50	0	0
AIIMS746	24	<50	<50	<50	<50	<50	<50	<50	<50	0	0

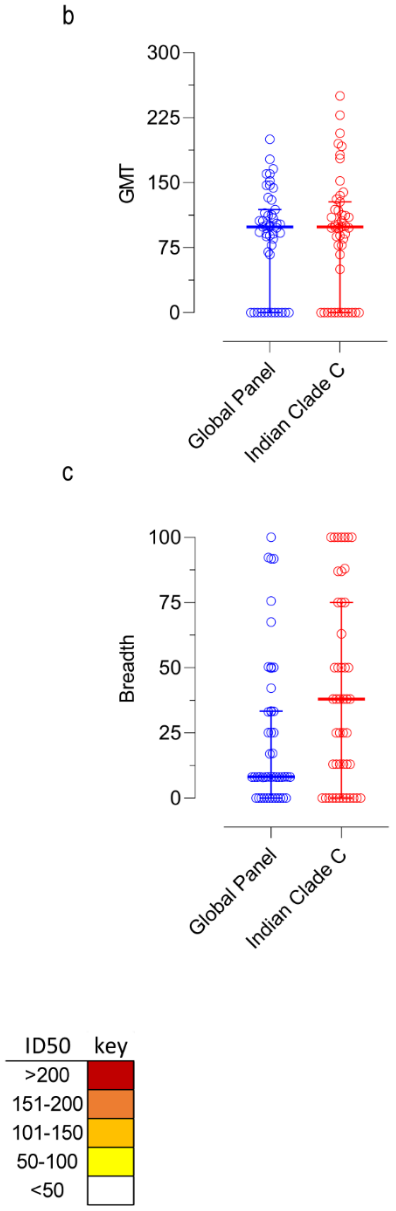

Supplementary Fig. 1 – Plasma Neutralization Activity of HIV-1 infected infants against viral isolates of Indian origin. (A) Heatmap representing HIV-1 specific neutralization titres (inverse plasma dilution) of plasma nAbs from 47 infant samples against the 8-virus Indian clade C panel. ID50 values are color-coded per the key given, with darker colors implying higher ID50 titres. (B and C) Comparison of geometric mean titres and breadth (pseudoviruses showing >50% neutralization at 1/50 plasma dilution) of infants (n=47) with the 12-virus global panel and 8-virus Indian clade C panel. All individual values are shown with lines drawn using median with interquartile range. Neutralization

assays were performed in triplicates and repeated thrice. Average ID50 values are shown and used for statistical comparisons.

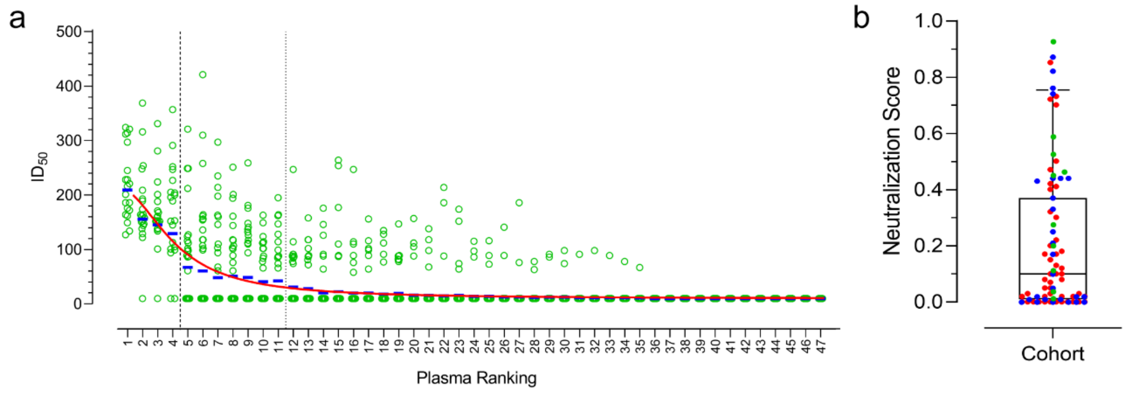

Supplementary Fig. 2 – Neutralization scores calculated based on modified breadth-potency matrix were predictive of geometric mean titres. (A) Infant plasmas are ranked based on geometric mean titres. Green dots represent individual ID50 values against the 12-virus global panel, whereas the blue bar represents geometric mean titres, red line shows non-linear curve fit of modified neutralization score. Neutralization scores accurately predict geometric mean titres. (B) Validation of neutralization score to predict elite and broad neutralizers {(red – infants (n=47), blue – adolescents (n=27) and green – adults (15)}. Quartile distribution of neutralization scores show 75th percentile at a cut-off of 0.35 and 90th percentile at 0.7. Elite neutralizers typically showed neutralization scores in the 90th percentile, whereas broad neutralizers had a neutralization score in the range of 75th to 90th percentile.

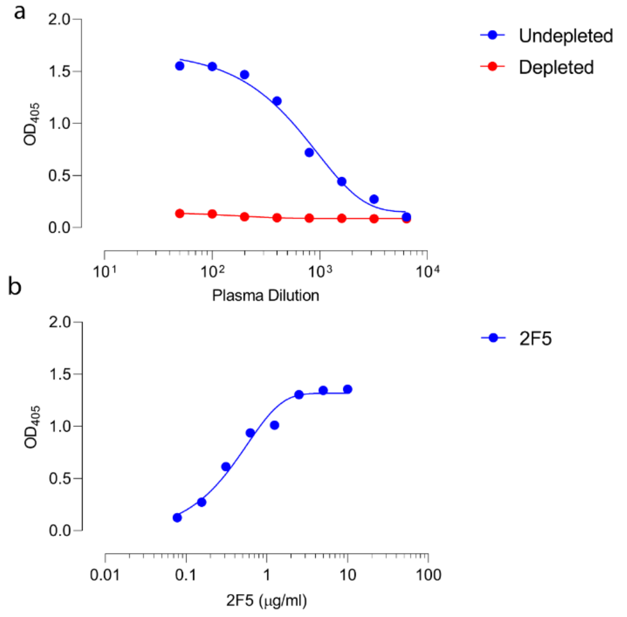

c

Virus	Clade	AIIMS704	Depleted
398F1	A	268	64
TRO11	B	141	122
X2278	B	152	112
25710	C	226	188
CE0217	C	386	<50
CE1176	C	115	78
X1632	G	265	56
246F3	AC	158	<50
CNE8	AE	161	<50
CNE55	AE	199	<50
BJOX2000	BC	264	<50
CH119	BC	176	<50
	GMT	198	95

ID50	key
>200	
151-200	
101-150	
50-100	
<50	

Supplementary Fig. 3 – AIIMS704 had MPER directed plasma bnAbs. (A) Binding ELISA against MPER-C peptide using undepleted and MPER-C depleted AIIMS704 plasma showed efficient adsorption and depletion of anti-MPER plasma bnAbs. Binding ELISAs were repeated thrice in duplicates and mean absorbance was used for plotting. (B) 2F5, an anti-MPER bnAb was used as positive control. (C) ID50 values of AIIMS704 undepleted and MPER-C depleted plasma against the 12-virus global panel. MPER depletion resulted in 2-fold reduction in GMT titres.

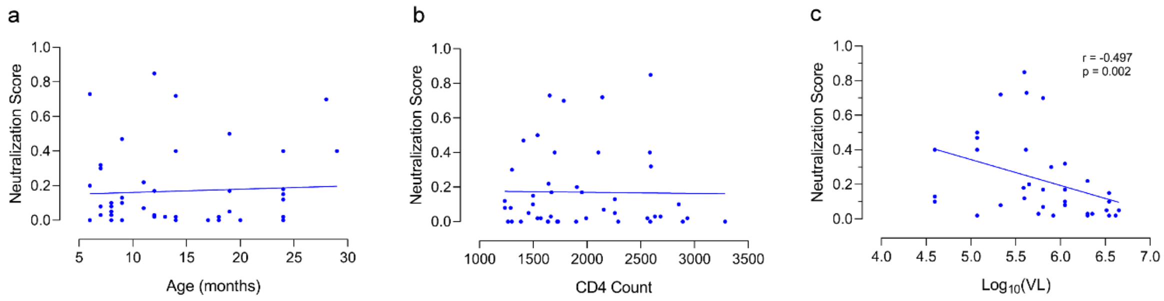

Supplementary Fig. 4 – Influence of host and viral parameter on neutralization breadth. Correlation between (A) duration of infection, (B) CD4 T-cell counts and (C) viral load with neutralization scores for infants (n=47) with discernible neutralization activity. Infants that showed no neutralization against the 12-virus global panel were excluded from analysis.

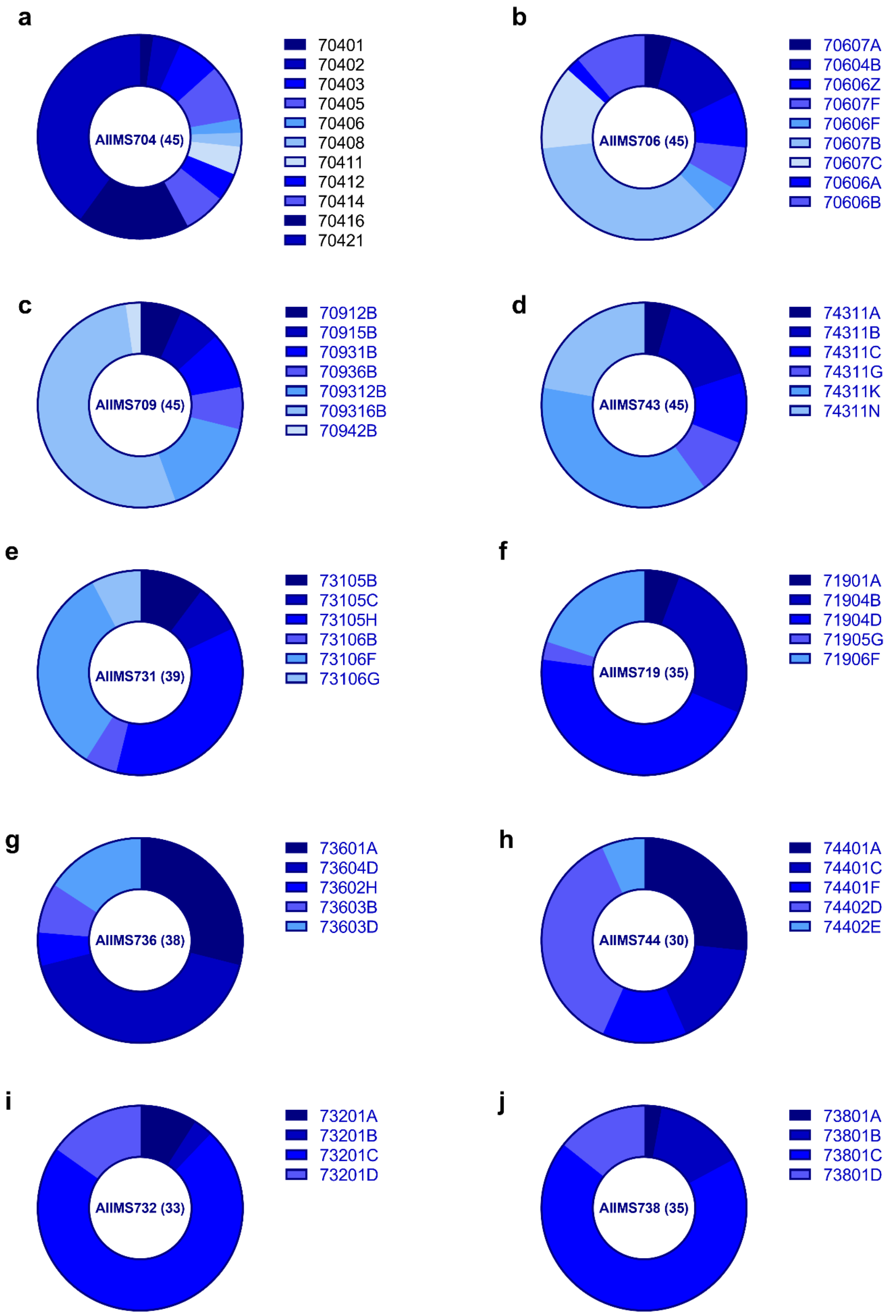

Supplementary Fig. 5 – Population frequency of SGA env amplicons from infant elite and broad neutralizers. (a-j) from top to bottom, left to right, donut plots show the frequency of SGA

amplicons of circulating viral variants in infant elite and broad neutralizers in the order of plasma ranking from figure 1. In the centre of each donut plots, infant ID with the number of total SGA amplicons in bracket is given. The frequency of individual clone in each infant is color coded, and is provided for each donut plot.

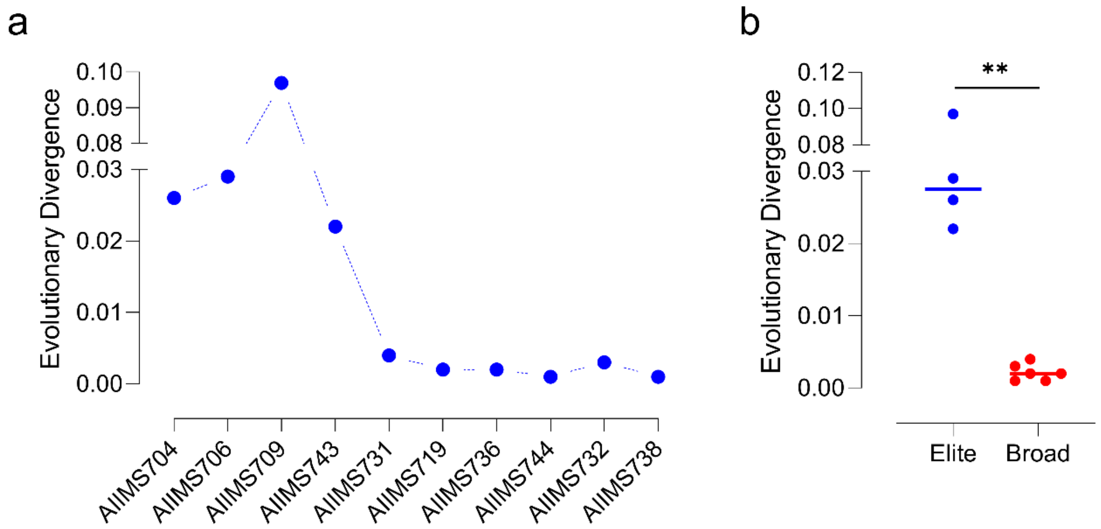

Supplementary Fig. 6 – Viral population in infant elite neutralizers is highly divergent. (A) Average evolutionary divergence (nucleotide substitutions per site) among SGA amplicons for infant elite (n=4) and broad (n=6) neutralizers. The number of base substitutions per site from averaging over all sequence pairs within each group are shown. Analyses were conducted using the Maximum Composite Likelihood model [1]. The rate variation among sites was modelled with a gamma distribution (shape parameter = 5). (B) Statistical comparison between elite and broad neutralizers was conducted by two-tailed Mann Whitney U test. ** represents a p-value of 0.0095.

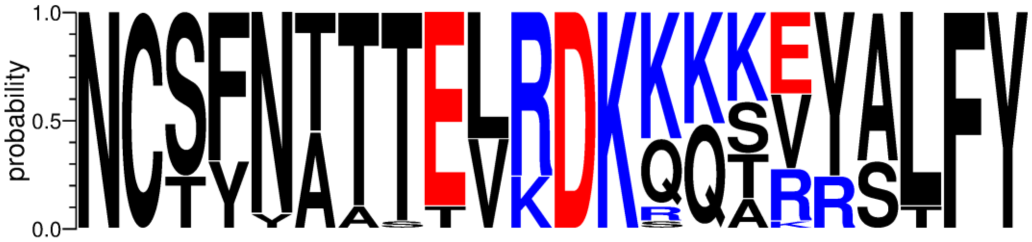

Supplementary Fig. 7 – Frequency plot of the V2-loop sequences of infant elite neutralizers. Weblogo plot showing the amino acid frequency plots of V2-loop (HXB2 amino acids 156 – 177) from all SGA amplicons of four infant elite neutralizers. Key glycan (N156 and N160) as well as lysine-rich strand B and C were well-conserved.

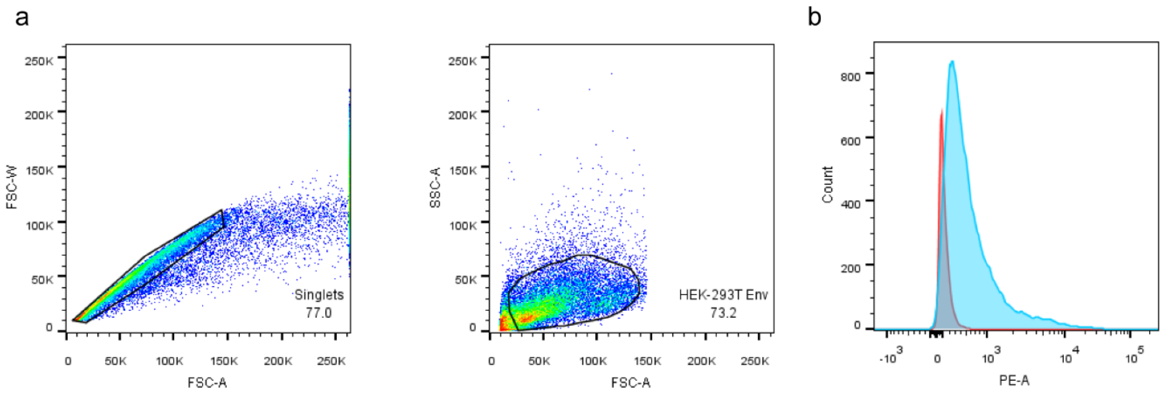

Supplementary Fig. 8 – Gating strategy for surface binding assay. (a) HEK293T cells transfected with individual Env from infant elite neutralizers were first gated for singlets followed by all cells and median fluorescence was calculated. (b) Histogram showing the intensity of phycoerythrin (PE) on non-transfected HEK293T (red) and Env transfected HEK293T (sky blue).
