# Supplementary material for: Broadly neutralizing plasma antibodies effective against autologous circulating viruses in infants with multivariant HIV-1 infection
Source: Nat Commun. 2020 Sep 2;11:4409. doi: 10.1038/s41467-020-18225-x (PMC7468291; doi:10.1038/s41467-020-18225-x)
Supplement: Supplementary file 3 — Reporting Summary [file 41467_2020_18225_MOESM3_ESM.pdf]

nature research

Corresponding author(s): Kalpana Luthra

Last updated by author(s): Jul 27, 2020

Reporting Summary

Nature Research wishes to improve the reproducibility of the work that we publish. This form provides structure for consistency and transparency in reporting. For further information on Nature Research policies, see our Editorial Policies and the Editorial Policy Checklist.

Statistics

For all statistical analyses, confirm that the following items are present in the figure legend, table legend, main text, or Methods section.

n/a	Confirmed	
☐	☒	The exact sample size (*n*) for each experimental group/condition, given as a discrete number and unit of measurement
☐	☒	A statement on whether measurements were taken from distinct samples or whether the same sample was measured repeatedly
☐	☒	The statistical test(s) used AND whether they are one- or two-sided<br>*Only common tests should be described solely by name; describe more complex techniques in the Methods section.*
☒	☐	A description of all covariates tested
☒	☐	A description of any assumptions or corrections, such as tests of normality and adjustment for multiple comparisons
☐	☒	A full description of the statistical parameters including central tendency (e.g. means) or other basic estimates (e.g. regression coefficient) AND variation (e.g. standard deviation) or associated estimates of uncertainty (e.g. confidence intervals)
☐	☒	For null hypothesis testing, the test statistic (e.g. F, t, r) with confidence intervals, effect sizes, degrees of freedom and P value noted<br>*Give P values as exact values whenever suitable.*
☒	☐	For Bayesian analysis, information on the choice of priors and Markov chain Monte Carlo settings
☒	☐	For hierarchical and complex designs, identification of the appropriate level for tests and full reporting of outcomes
☒	☐	Estimates of effect sizes (e.g. Cohen's d, Pearson's r), indicating how they were calculated

Our web collection on statistics for biologists contains articles on many of the points above.

Software and code

Policy information about availability of computer code

Data collection	No software was used.
Data analysis	GraphPad Prism 8.3, MEGA X 10.1, Sequencher 5.4, PhyML 3.1, RIP 3.0, HIV-1 Database Webtools (Highlighter, AnalyzeAlign)

For manuscripts utilizing custom algorithms or software that are central to the research but not yet described in published literature, software must be made available to editors and reviewers. We strongly encourage code deposition in a community repository (e.g. GitHub). See the Nature Research guidelines for submitting code & software for further information.

Data

Policy information about availability of data

All manuscripts must include a data availability statement. This statement should provide the following information, where applicable:

- Accession codes, unique identifiers, or web links for publicly available datasets
- A list of figures that have associated raw data
- A description of any restrictions on data availability

The SGA amplified HIV-1 envelope sequences used for inference of phylogeny and highlighter plots are available at GenBank with accession numbers MN703343 – MN703404 and MT366192 – MT366197. All data required to state the conclusions in the paper are present in the paper and/or the supplementary data. Source data are provided with this paper. Additional information related to the paper, if required, can be requested from the authors.

Field-specific reporting

Please select the one below that is the best fit for your research. If you are not sure, read the appropriate sections before making your selection.

☒ Life sciences ☐ Behavioural & social sciences ☐ Ecological, evolutionary & environmental sciences

For a reference copy of the document with all sections, see nature.com/documents/nr-reporting-summary-flat.pdf

Life sciences study design

All studies must disclose on these points even when the disclosure is negative.

Sample size	In this descriptive observational study, no sample size calculation was performed. We recruited all HIV-1 infected infants that visited the Pediatric Chest Clinic, Department of Paediatrics, All India Institute of Medical Sciences during the duration of the study to identify factors responsible for early induction of bnAbs in infants.
Data exclusions	From the 51 HIV-1 infected infants recruited in this study, four infants were excluded based on the reactivity of their plasma neutralizing antibodies against murine leukemia Env pseudotyped viruses (non-specific neutralization) and were not characterized further. Data from the remaining 47 infants was used in all analysis.
Replication	Neutralization assays were performed in triplicates and repeated thrice. Average ID50 values are shown and used for statistical comparisons. Binding ELISAs were performed in duplicates and repeated thrice. Average OD450 values were used for plotting curves. Surface binding assay was performed thrice and average PE-MFI (phycoerythrin-median fluorescence intensity) values were used for plotting curves. The same source material (plasma sample) was used in all replicates. Data from all replicates are included in the analysis. All attempts at replication were successful.
Randomization	The study was observational and not randomized. HIV-1 infected infants were allocated into elite, broad and cross-neutralizer groups based on their neutralization activity against the 12-virus global panel.
Blinding	No blinding was performed. The study was aimed to identify viral factors associated with the development of broadly neutralizing antibody responses in HIV-1 infected infants.

Reporting for specific materials, systems and methods

We require information from authors about some types of materials, experimental systems and methods used in many studies. Here, indicate whether each material, system or method listed is relevant to your study. If you are not sure if a list item applies to your research, read the appropriate section before selecting a response.

Materials & experimental systems

n/a	Involved in the study	
☐	☒	Antibodies
☐	☒	Eukaryotic cell lines
☒	☐	Palaeontology and archaeology
☒	☐	Animals and other organisms
☐	☒	Human research participants
☒	☐	Clinical data
☒	☐	Dual use research of concern

Methods

n/a	Involved in the study	
☒	☐	ChIP-seq
☐	☒	Flow cytometry
☒	☐	MRI-based neuroimaging

Antibodies

Antibodies used	bnAbs used in this study were (clone name/ and source reference): PG9 (Walker 2009 Science/19729618), PG16 (Walker 2009 Science/19729618), PGT145 (Walker 2011 Nature/21849977), PGDM1400 (Sok PNAS 2014/25422458), CAP256.09 (Doria-Rose 2014 Nature/24590074), CAP256-.25 (Doria-Rose 2016 J Virol/26468542), 10-1074 (Mouquet 2012 PNAS/23115339), BG18 (Freund 2017 Sci Transl Med/28100831), AIIMS-P01 (Kumar 2018 J Virol/30429339), PGT121 (Walker 2011 Nature/21849977), PGT128 (Walker 2011 Nature/21849977), PGT135 (Walker 2011 Nature/21849977), VRC01 (Wu 2010 Science/20616233), N6 (Huang 2016 Immunity/27851912), 3BNC117(Scheid 2011 Science/21764753), PGT151 (Falkowska 2014 Immunity/24768347), 35O22 (Huang 2014 Nature/25186731), 10E8 (Huang 2012 Nature/23151583), 4E10 (Stiegler 2001 AIDS Res Hum Retroviruses/11788027). non-nAbs used in this study were(clone name/ and source reference): b6 (delReal 1999 Mol Immunol/10593511), F105 (Posner 1992 J Immunol/1710248), 17b (Rizutto 1998 Science/9632396), 48d (Thali 1993 J Virol/7685405), A32 (Moore 1993 J Virol/7687303), 447-52D (Buchbinder 1992 AIDS Res Hum Retroviruses/1466965). Commercial antibodies used (supplier/catalog number): HRP-conjugated Goat anti-Human IgG Secondary Antibody (Invitrogen/62-8420), PE anti-human IgG Fc Antibody (Biolegend/409304).
Validation	All bnAbs and non-nAbs were procured from NIH AIDS Reagent Program and Neutralizing Antibody Consortium, International AIDS Vaccine Initiative. As the antibody preparations used in the current study were from different sources we validated their performance against the 12-virus global panel used for neutralization assays for plasma nAbs from HIV-1 infected infants. Observed

activities of all Ab preparations matched the reported activity of the respective bnAbs against the 12-virus global panel available at HIV-1 Database (see CATNAP; https://www.hiv.lanl.gov/components/sequence/HIV/neutralization/main.comp), and were thus rated as valid.

Eukaryotic cell lines

Policy information about cell lines

Cell line source(s)	HEK293T cells (American Type Culture Collection (ATCC)). TZM-bl cells (NIH AIDS Reagent Program)
Authentication	No specific authentication was performed. Both cell lines have been routinely used in our laboratory for years. Cell cultures are restarted from freezer stocks in 1-2 months.
Mycoplasma contamination	All used cell stocks tested negative for mycoplasma.
Commonly misidentified lines (See ICLAC register)	No commonly misidentified cell lines were used

Human research participants

Policy information about studies involving human research participants

Population characteristics	A total of 51 HIV-1 infected infants (29 males/22 females) were recruited. The median age for infected infants was 12-months (IQR, 8 – 19), the median CD4 count was 1731 cells/mm3 (IQR, 1498 – 2562) and the median viral load on log scale was 5.804 RNA copies/ml (IQR, 5.331 – 6.301).
Recruitment	Antiretroviral naïve and asymptomatic HIV-1 infected infants below the age of 2-years visiting the Pediatric Chest Clinic, Department of Pediatrics, AIIMS during the duration of this study were recruited randomly. A total of 51 antiretroviral naïve and asymptomatic HIV-1 infected infants were recruited for this study. Infants were recruited randomly, regardless of gender, to avoid any self-selection bias.
Ethics oversight	The study was approved by institute ethics committee of All India Institute of Medical Sciences (IECPG-307/07.09.2017).

Note that full information on the approval of the study protocol must also be provided in the manuscript.

Flow Cytometry

Plots

Confirm that:

- [x] The axis labels state the marker and fluorochrome used (e.g. CD4-FITC).
- [x] The axis scales are clearly visible. Include numbers along axes only for bottom left plot of group (a 'group' is an analysis of identical markers).
- [x] All plots are contour plots with outliers or pseudocolor plots.
- [x] A numerical value for number of cells or percentage (with statistics) is provided.

Methodology

Sample preparation	1.25 x 10^5 HEK293T cells seeded in a 12-well plate were transiently transfected with 1.25 µg of env-coding plasmids (pcDNA3.1 with cloned env/rev cassettes) using PEI-MAX. 48 hours post-transfection, cells were harvested and per experimental requirement, distributed in 1.5 ml microcentrifuge tubes. For sCD4 triggering, 10 µg/ml of 2-domain sCD4 was added and incubated for 30 minutes at room temperature. For monoclonal antibody staining, 10 µg/ml of antibody was used and titrated 2-fold in staining buffer. 100 µl of primary antibody (HIV-1 specific monoclonals) were added to HEK293T cells expressing envs, and incubated for 30 minutes at room temperature. After washing, 100 µl of 1:500 diluted PE conjugated goat anti-human Fc was added, and after 30-minute incubation, a total of 50,000 cells were acquired on BD LSRFortessa X20.
Instrument	BD LSRFortessa X20
Software	FlowJo v10.6.1
Cell population abundance	HEK293T cell cultures transfected with HIV-1 envelope glycoprotein were used for flow cytometry analysis.
Gating strategy	HEK293T cell cultures were used and, therefore, no specific gating was required. All cells were analyzed for expression of HIV-1 envelope glycoprotein as described in figure 5.

- [x] Tick this box to confirm that a figure exemplifying the gating strategy is provided in the Supplementary Information.
